# Neurodegeneration Beyond Neurons: Emerging Insights from Molecular and Multi-Omics Approaches

**DOI:** 10.3390/biomedicines14030635

**Published:** 2026-03-12

**Authors:** Marta Lualdi

**Affiliations:** Department of Science and High Technology, Center of Research in Neuroscience, University of Insubria, 21052 Busto Arsizio, VA, Italy; marta.lualdi@uninsubria.it

## 1. The Current State of Molecular Neuroscience

Biological systems are inherently complex, with multiple components that interact with each other. The behavior of the system cannot be summarized and interpreted as the result of a simple sum of all components, and system alterations represent the basis on which pathologies arise [1].

Neuroscience represents a rapidly evolving research field, in which basic cellular and molecular studies, clinical observations, and bioinformatics must be efficiently integrated to deepen our understanding of physiological and pathological processes [2]. The neurodegenerative disease (ND) category encompasses both distinct and partially overlapping pathological phenotypes in which genetic and environmental factors typically contribute to the onset and progression, and aging represents one major risk factor (at least in the context of sporadic forms) [3,4]. NDs are typically diagnosed late after disease onset because of the lack of early diagnostic biomarkers and shared diagnostic criteria. Most therapeutic strategies focus on symptom reduction and aim at a general improvement in life conditions, but they cannot halt disease progression or be considered disease-modifying treatments. In addition, huge variability is typically observed in terms of both clinical manifestations and response to pharmacological therapies. Thus, in aging populations, the increasing prevalence of NDs represents a huge problem not only for the healthcare system but also for society and economics.

In this context, the major challenge in neurodegeneration research is finding strategies that can improve early diagnosis, as well as patient classification and stratification, thus contributing to the development of novel, effective therapeutic approaches. One main objective is to determine the complex network of cellular and molecular mechanisms that trigger disease onset and foster its progression.

Canonical targeted research strategies, which are hypothesis-driven and focus on a single or a few aspects of the disease, have been successfully used in the past to identify fundamental underlying pathological mechanisms. Prominent examples are the mechanisms of aberrant proteostasis (protein aggregation), oxidative stress, mitochondrial dysfunction, and alterations of vesicle dynamics in the two most frequent NDs worldwide, i.e., Alzheimer’s disease (AD) and Parkinson’s disease (PD) [5,6]. However, targeted approaches alone are now unlikely to be sufficient to drive major advances in ND research. However, omics technologies (e.g., genomics, transcriptomics, proteomics, and metabolomics) are promising strategies for studying disease complexity, as well as the use of bioinformatic tools for feature selection and omics data integration, within a systems biology framework [7,8].

## 2. Special Issue “Cellular and Molecular Biology of Neurodegenerative Disorders”

When I was invited to serve as a Guest Editor of the Special Issue (SI) entitled “Cellular and Molecular Biology of Neurodegenerative Disorders” in *Biomedicines*, I was enthusiastic about contributing to the growing body of knowledge on mechanisms underlying neurodegeneration. The articles included in this SI collectively portray neurodegeneration as a multifactorial and system-level process in which intrinsic neuronal vulnerability intersects with systemic, environmental, and therapeutic factors.

At the cellular level, mitochondrial dysfunction, oxidative stress, protein dyshomeostasis, and dysregulated intracellular signaling are core determinants of neuronal cells fragility [9,10,11]. Khodyreva et al. [12] underlined that efficient DNA repair is also essential for neuronal survival and described how its dysregulation contributes to neurons vulnerability and cell death. This offers novel molecular targets for intervention in NDs in addition to those already developed based on the molecular mechanisms driving the degeneration of neuronal cells.

The discovery of neuron-extrinsic modulators highlights how peripheral and environmental factors converge on shared molecular cascades, ultimately reinforcing neuron-intrinsic pathogenic pathways, such as protein aggregation, synaptic dysfunction, and inflammation. In this context, gut-microbiota-derived metabolites are attracting increasing attention in ND research [13,14,15]. Munteanu et al. [16] demonstrated that microbiota-derived metabolites are key modulators of neurodegenerative processes. In particular, hydrogen sulfide appears to exert dual roles in both AD and PD: protective at physiological levels but potentially damaging when dysregulated, contributing to neuroinflammation, amyloid-beta accumulation, and alpha-synuclein aggregation. Thus, metabolic rebalancing of the gut ecosystem may represent a novel therapeutic avenue. In addition to the role of the microbiota, systemic metabolic dysfunction can also play a role in NDs. Mahon et al. [17] demonstrated how altered metabolism induces brain molecular alterations reminiscent of neurodegeneration, further underscoring the importance of metabolic–neural crosstalk. In addition, the role of specific traumas in initiating and promoting NDs is an interesting topic under investigation [18,19,20]. Zedde et al. [21] revised how traumatic brain injury (TBI) may initiate long-term pathological cascades that converge with classical neurodegenerative pathways, such as amyloid deposition and tau pathology, reinforcing the concept of neurodegeneration as a multifactorial and overlapping process.

Another central aspect in ND research, which is also the main goal of most multi-omics studies involving large cohorts of patients, is the identification and exploitation of reliable disease biomarkers. Beyond diagnostic utility (early diagnosis), biomarkers are increasingly important for patient stratification, prognosis, prediction of therapeutic outcomes, and pharmacodynamic monitoring [22,23]. Maretina et al. [24] underlined why robust biomarkers (from molecular, functional, and imaging studies) are essential for precision medicine approaches, which are required to increase treatment effectiveness for spinal muscular atrophy (SMA). This is also relevant for other (if not all) NDs.

Finally, in the context of the available therapeutic strategies, the neuromodulatory interventions originally developed to control disease symptoms may extend beyond symptomatic control toward neuroprotection. Nag et al. [25] underscored that deep brain stimulation (DBS) may exert long-term neuroprotective effects at the cellular and molecular levels in patients with PD, involving brain-derived neurotrophic factor (BDNF) signaling, mitochondrial function, oxidative stress reduction, inflammation modulation, and synaptic plasticity. Notably, this supports a disease-modifying effect of DBS, with a therapeutic potential broader than neuronal circuitry modulation.

Collectively, this SI gathered studies that advocate for an integrated view of NDs in order to uncover novel pathological mechanisms and indicate directions for developing disease-modifying therapeutic interventions. This ultimately requires a shift from single-pathway models toward multi-omics, systems biology, and precision medicine.

## 3. Conclusions and Perspectives

The recent literature in the neurodegeneration research field strongly suggests that NDs should no longer be viewed as purely neuron-centered diseases but rather as complex, multi-level processes shaped by the interactions between intrinsic neuronal vulnerability and systemic modulators. This implies that deciphering neurodegeneration will increasingly rely on integrative approaches combining multi-omics technologies, systems biology, and advanced biomarker discovery platforms. Such frameworks will be essential for translating molecular knowledge into early disease detection, patient stratification strategies, and into more effective disease-modifying therapies.

Ultimately, progress in this field will depend on the development of large, multidisciplinary consortia able to coordinate extensive patient recruitment as well as generate and integrate large-scale datasets. Such collaborative frameworks will be essential for applying analytical approaches capable of navigating data dimensionality and extracting biologically meaningful and clinically actionable knowledge.
